# Molecular functions and therapeutic applications of exosomal noncoding RNAs in cancer

**DOI:** 10.1038/s12276-022-00744-w

**Published:** 2022-03-29

**Authors:** Qin-Wen Liu, Yan He, Wen Wen Xu

**Affiliations:** Institute of Biomedicine, Guangdong Provincial Key Laboratory of Bioengineering Medicine, National Engineering Research Center of Genetic Medicine, Jinan University, Guangzhou, 510632 China

**Keywords:** Cancer, Non-coding RNAs

## Abstract

Cancer is one of the most difficult diseases in human society. Therefore, it is urgent for us to understand its pathogenesis and improve the cure rate. Exosomes are nanoscale membrane vesicles formed by a variety of cells through endocytosis. As a new means of intercellular information exchange, exosomes have attracted much attention. Noncoding RNAs exist in various cell compartments and participate in a variety of cellular reactions; in particular, they can be detected in exosomes bound to lipoproteins and free circulating molecules. Increasing evidence has suggested the potential roles of exosomal noncoding RNAs in the progression of tumors. Herein, we present a comprehensive update on the biological functions of exosomal noncoding RNAs in the development of cancer. Specifically, we mainly focus on the effects of exosomal noncoding RNAs, including microRNAs, circular RNAs, long noncoding RNAs, small nuclear RNAs, and small nucleolar RNAs, on tumor growth, metastasis, angiogenesis, and chemoresistance. Moreover, we outline the current clinical implications concerning exosomal noncoding RNAs in cancer treatment.

## Cancer: regulatory RNAs in tiny packages

RNA molecules carried in small membrane-bound vesicles called exosomes, which are released naturally by some cells, are implicated in controlling the development of some cancers and may offer new approaches to treatment. Wen Wen Xu and colleagues at Jinan University in Guangzhou, China review the functions of small RNAs carried by exosomes in the development of cancer. They focus on a variety of ‘noncoding’ RNAs, which do not determine the manufacture of protein molecules but instead perform genetic regulatory functions. Some RNAs support the progression of specific cancers while some suppress other cancers. Their presence could perhaps be used to diagnose and predict the progress of particular cancers. The emerging research findings suggest exosomes might be used to treat cancer by delivering small regulatory RNA molecules into tumors, perhaps with other drugs.

## Introduction

Since the twenty-first century, the incidence rate of cancer has increased at an alarming rate along with the changing lifestyles and habits and increased life expectancy of people. Current thinking suggests that cancer is a complicated disease caused by accumulating genomic and epigenetic aberrations^1^. There are eight biological capabilities acquired during the multistep development of human tumors to rationalize the complexities of neoplastic disease^2^. These processes are involved in sustaining proliferative signaling, evading growth suppressors, resisting cell death, enabling replicative immortality, inducing angiogenesis, activating invasion and metastasis, reprogramming energy metabolism, and evading immune destruction. Therefore, it is imperative to better understand the mechanisms of tumorigenesis and development to improve therapeutic efficacy.

Extracellular vesicles (EVs) are structures released by all types of cells into the environment^3^. They are enveloped by a phospholipid bimolecular layer and can be composed of a variety of components. Exosomes, a subtype of EVs, can deliver proteins, nucleic acids and noncoding RNAs (ncRNAs) to recipient cells for intercellular communication. Since certain proteins and nucleic acids can be transferred to receptor cells in the tumor microenvironment (TME), exosomes can function to transform healthy cells into cells with the characteristics of cancer cells and create a fertile environment for tumor cells to grow^4^. Currently, studies on the impact of exosomes on tumorigenesis and carcinoma progression have expanded^5–7^. In addition, due to the stability of exosomes and their low immunogenicity, exosomes have been developed as vehicles to carry drugs and antitumor nucleic acids for tumor therapy.

Approximately 50% of the DNA in the genome can be transcribed into RNA, of which only 2% can be translated into proteins, and the remaining 98%, known as ncRNAs, cannot be translated, including small nuclear RNAs (snRNAs), small nucleolar RNAs (snoRNAs), circular RNAs (circRNAs), microRNAs (miRNAs), piwi-interacting RNAs (piRNAs), long noncoding RNAs (lncRNAs) and small interfering RNAs (siRNAs). Through interaction with DNA, RNA, and proteins, ncRNAs can modulate the expression levels of genes at the transcriptional, posttranscriptional, and epigenetic levels, thus determining the fate of cells^8,9^. In this review, we mainly provide an overview of the roles of exosomal ncRNAs, especially exosomal miRNAs, exosomal circRNAs, and exosomal lncRNAs, in tumor progression, including tumor growth, metastasis, angiogenesis, and chemoresistance. At the same time, we briefly summarize the current application of exosomal ncRNAs in tumor therapy.

## The biological characteristics of exosomes

### The structure and biogenesis of exosomes

When the cell is activated or damaged, some of the proteins, nucleic acids, lipids, and other substances will be separated into multivesicular bodies (MVBs) and released to the extracellular matrix. According to the diameter, MVBs can be divided into three types^10^: (1) exosomes with a diameter of 30–150 nm; (2) microparticles with a diameter of 100–1000 nm; and (3) apoptotic bodies with a diameter of 1–4 μm^11^. Initially, exosomes were described by Trams et al. as exfoliated vesicles^12^, and then Harding and Stahl discovered them in rat reticulocytes^13^. Finally, Johnstone named these structures “exosomes” in 1987^14^. Due to the lack of research methods, exosomes were once considered “garbage bags” produced by host cells to maintain the balance of cell components and secrete intracellular waste products without any biological functions^14^. Upon in-depth study of exosomes, however, it has been confirmed that mast cells, dendritic cells, lymphocytes, fibroblasts, mesenchymal stem cells, and tumor cells can all produce and release exosomes. A growing body of evidence has revealed that these “unnecessary compounds” actually have an important impact on physiological and pathological processes, such as damage repair^15^, antigen presentation^16^, tumor metastasis, and other important pathways^17^.

**Fig. 1:**
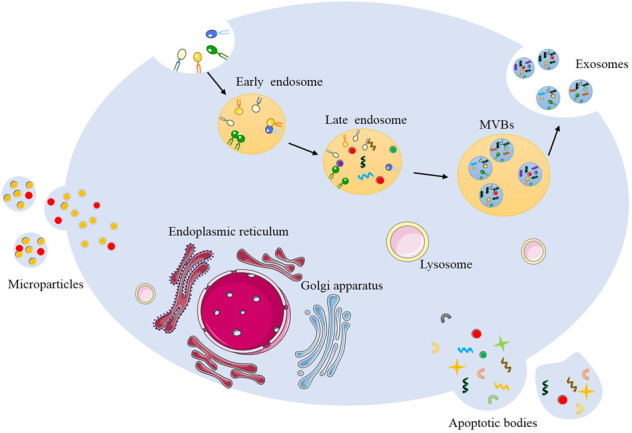
The biological characteristics of exosomes, microparticles, and apoptotic bodies. Exosome biogenesis begins with the inward budding of the plasma membrane and then the formation of early endosomes. With the assistance of the ESCRT protein family, a variety of DNA, RNA, and proteins gather to form late endosomes, further developing MVBs. Finally, MVBs are released into the extracellular environment by fusing with the plasma membrane to form exosomes. Both microparticles and apoptotic bodies arise through the extension of the plasma membrane.

Exosomes are secreted in different ways from the other two MVBs^18^, as shown in Fig. 1. Both microparticles and apoptotic bodies are produced through the extension of the plasma membrane, while exosomes are produced in the form of intraluminal vesicles (ILVs). In the early stage of exosome formation, the plasma membrane buds inward and forms ILVs, also known as early endosomes. The endosomal sorting complex required for transport (ESCRT) protein family is responsible for recruiting cluster-specific substances and transporting them to the endosomal membrane. Four subtypes of ESCRT proteins, ESCRT0-III, work together to form late endosomes^19^. With the accumulation of cargoes and inward budding of endosome membranes, multiple ILVs containing proteins, RNA, and other substances are formed. Presently, they are called MVBs. MVBs can be used as transporters to deliver cargoes to lysosomes for degradation, or they can fuse with the plasma membrane through the regulation of Rab proteins to release ILVs, which are exosomes. Of course, there are various other regulators involved in the complex process of exosome formation^20^.

### The composition of exosomes

Biological methods such as differential centrifugation, sucrose gradient ultracentrifugation, transmission electron microscopy, western blotting, and mass spectrometry are used to analyze the composition and function of exosomes. Their contents include proteins, DNA, RNA, and even genetic materials of viruses and prions. Among them, proteins can be divided into specific proteins and nonspecific proteins. Most nonspecific proteins are derived from cytoplasmic and membrane proteins in the conserved regions of parental cells, which mainly maintain the structure and function of exosomes. Specific proteins are related to the origin of parent cells, such as exosomes from T lymphocytes with granular enzymes and perforin proteins on the surface^21^.

In addition to proteins, exosomes contain a large number of RNAs that can perform biological functions^22^. NcRNAs are biological molecules that cannot encode proteins (i.e., they have no protein-coding function) but can control gene expression and cell differentiation at the genomic and chromosomal levels. In recent decades, researchers have realized that ncRNAs are no longer “junk” transcriptional products but are biological molecules with regulatory functions that mediate various biological processes. Many ncRNAs can be recruited by ESCRT proteins to form exosomal ncRNAs. Through deep sequencing analysis, the ncRNAs identified from exosomes in human plasma and urine included snRNAs, snoRNAs, circRNAs, miRNAs, piRNAs, lncRNAs, ribosomal RNAs (rRNAs), and transfer RNAs^23,24^. In comparison with free ncRNAs, exosomal ncRNAs, as a new vector, can not only protect ncRNAs from enzyme degradation but also deliver ncRNAs to produce targeted biological effects^25^. We do not fully understand how exosomal ncRNAs exert their function in different tumors thus far, but there is no doubt that they play a crucial role in tumor progression. Exosomes are a double-edged sword, and in-depth research will pave the way for accurate and effective treatments.

## The roles of exosomal miRNAs in cancer

### Background on miRNAs

MiRNAs refer to single-stranded RNA molecules approximately 22 nucleotides in length that mainly regulate gene expression at the posttranscriptional level. MiRNAs can not only reduce the protein produced by transcripts but also reduce the level of the transcripts^26^. A single miRNA can target hundreds of mRNAs and influence the expression of target genes that are often involved in functional interacting pathways. In fact, many diseases are associated with unique miRNA characteristics, which indicates that specific miRNAs are activated in some pathophysiological processes^27^. The TME, where exosomes are released, is critical for the malignant phenotypes of tumors. Valadi et al. showed early on that exosomal miRNAs could be delivered to recipient cells to program relevant functions^5^. Moreover, Umezu et al. found that miRNAs mediated communication between neoplasia and endothelial cells^28^. Similarly, it has also been demonstrated that miRNAs are transferred from monocytes to endothelial cells and modulate endothelial cell function^29^. In the last decade, multiple studies have revealed that cancer-derived exosomal miRNAs play important roles in the recruitment and reprogramming of constituents of the tumor environment^30^. Since miRNAs are important gene expression regulators and candidate genes for the development of biomarkers, research on miRNAs is rapidly expanding.

### Exosomal miRNAs in tumor growth

Tumor growth is a malignant phenotype of tumors; thus, increasing attention has been given to understanding the mechanisms of cell proliferation. Advances in miRNA research provide new opportunities for cancer treatment^31,32^. Our previous studies have shown that higher miR-377 expression is related to the progression of esophageal squamous cell carcinoma (ESCC), suggesting a novel direction for the treatment and diagnosis of ESCC^33^. Increasing studies have recently suggested a relationship between exosomal miRNAs and tumor growth. For example, a recent study on esophageal adenocarcinoma demonstrated that decreased levels of miR-30a-3p and miR-30a-5p were associated with the activation of the Wnt signaling pathway, enhancing cell proliferation and tumor growth^34^. In another study of hepatocellular carcinoma (HCC), cancer cells were shown to secrete nanovesicles and exosomes, which were different from their source cells^35^. In addition, exosomal miR-126 from endothelial cells was found to be related to the progression of non-small-cell lung cancer (NSCLC) cells and malignant mesothelioma^36,37^. Similarly, in ovarian cancer (OC), exosomal miRNA-454 was suggested to disrupt the Wnt pathway by targeting proline-rich transmembrane protein 2, thereby promoting OC cell growth in vivo^38^. Intriguingly, Melo et al. reported that breast cancer (BC)-derived exosomes contain miRNAs associated with the RISC-Loading Complex, which has the cell-independent capacity to process precursor miRNAs into mature miRNAs, thus promoting tumorigenesis^39^.

### Exosomal miRNAs in tumor metastasis

Metastasis is an overwhelming cause of mortality in cancer patients. Unsurprisingly, exosomal miRNAs are also involved in tumor metastasis. Cancer-secreted miRNAs are emerging mediators of cancer-host crosstalk. For example, highly metastatic HCC cell-secreted exosomal miR-1247-3p directly targets B4GALT3 to activate β1-integrin-NF-κB signaling in fibroblasts, thus increasing the ability of normal fibroblasts to transform into CAFs^40^. In addition, exosomal miRNA-105, which is characteristically expressed and secreted by metastatic BC, has been shown to promote the metastasis of BC by targeting the tight junction protein ZO-1^41^. Similarly, high miR-122 levels in the circulation have been proven to be associated with metastasis in BC^42^. In addition, miR-19a and miR-19b have been reported to be associated with the progression of liver metastasis in human colorectal cancer (CRC)^43^. One study suggested that the expression of 11 exosomal miRNAs in patients with metastatic PC changed significantly compared with that in patients with nonmetastatic PC, of which miR-375 and miR-200b were confirmed to be related to metastasis^44^. Another study on PC also showed a significant increase in exosomal miRNA-141 levels in patients with metastatic features^45^.

### Exosomal miRNAs in tumor angiogenesis

Angiogenesis, the development of new blood vessels from preexisting vessels, plays an important role in tumor growth, maintenance and metastasis^46^. Increasing evidence suggests the potential role of exosomal miRNAs in tumor angiogenesis. For example, exosomal miR-9 has been found to effectively reduce SOCS5 levels and activate the JAK-STAT pathway, further promoting endothelial cell migration and tumor angiogenesis^47^. MiR-210 derived from exosomes was proven to be enriched in exosomes targeted to stromal cells. Upon further study, Cui et al. found that exosomal miR-210 reduces the expression of the miR-210 target protein Ephrin A3, thereby promoting lung adenocarcinoma angiogenesis^48^. Additionally, another study on exosomal miR-210 mediated by neutral sphingomyelinase 2 (nSMase2) suggested that it can be transported to endothelial cells and participate in the suppression of target genes, thereby enhancing angiogenic activities^49^. Moreover, a study showed that exosomal miR-21 leads to the activation of STAT3, which elevates the level of VEGF in human bronchial epithelial cells and is involved in the angiogenesis of human umbilical vein endothelial cells (HUVECs)^50^. Similarly, miR-221-3p derived from exosomes was found to be significantly enriched in cervical squamous cell carcinoma^51^.

### Exosomal miRNAs in tumor chemoresistance

Chemoresistance challenges the clinical outcomes of cancer patients and remains the main obstacle to cancer therapy^52^. Recent studies have suggested that some miRNAs are likely to be responsible for drug resistance in various cancers. Camptothecin is a drug used in cancer chemotherapy to induce apoptosis. However, analyses have shown that overexpression of exosomal miR-373 inhibits the camptothecin-induced apoptosis of tumor cells^53^. In addition, the chemotherapy drug cisplatin has long been one of the first-line drugs for fighting tumors, especially those of the lung, ovary, and testes. Challagundla et al. found that exosomal miR-155 transferred by human monocytes can immediately target telomeric repeat binding factor 1 (TERF1) and elevate neuroblastoma cell resistance to cisplatin^54^. Likewise, 5-fluorouracil (5-FU) is also a crucial agent for adjuvant therapy after curative surgery^55–57^. A study showed that in the serum exosomes of drug-resistant CRC patients, the expression levels of some miRNAs, such as miR-21-5p and miR-1246, were significantly higher than those in the serum exosomes of sensitive control patients. The authors pointed out that targeting these miRNAs could promote the chemotherapy sensitivity of CRC cells, which may provide a new direction for CRC therapy^58^. However, miR-29c was shown to be frequently downregulated in the serum samples of patients with ESCC, which has also been shown to be related to the resistance of ESCC to 5-FU^59^.

**Fig. 2:**
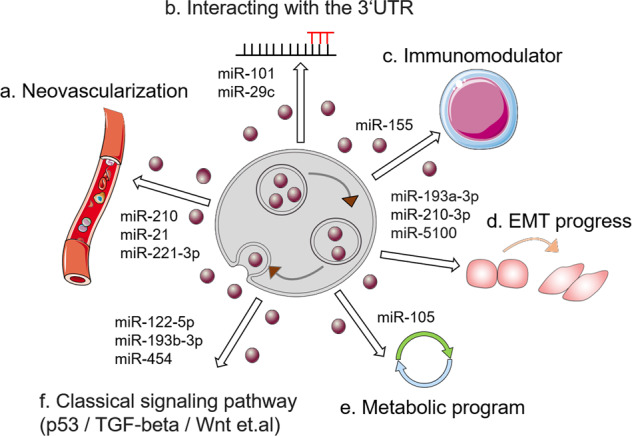
The mechanisms of the exosomal miRNAs involved in cancer. **a** Promoter angiogenesis. **b** Interacting with the 3′ UTR of its target genes and disrupting its transcription level. **c** Regulating the immune system as immunomodulators. **d** Influencing the progress of EMT. **e** Participating in tumor metabolic reprogramming. **f** Affecting the classic signaling pathways involved in tumorigenesis and development.

In general, the mechanism by which exosomal miRNAs promote tumor progression can be divided into the following categories: (a) Regulating the expression of genes involved in angiogenesis, such as VEGF^60,61^ (Fig. 2a); (b) Direct binding to the 3′ UTR of the target gene, thus affecting its function^62,63^ (Fig. 2b); (c) Interfering with the immune system and disrupting its function^64,65^ (Fig. 2c); (d) Participating in the EMT process^66,67^ (Fig. 2d); (e) Reprogramming metabolic balance^68,69^ (Fig. 2e); (f) Activating classical signaling pathways related to tumorigenesis and development^70,71^ (Fig. 2f). The corresponding exosomal miRNAs in each mechanism are shown as representatives in Fig. 2(a–f).

## The roles of exosomal circRNAs in cancer

### Background on circRNAs

CircRNAs are a novel class of ncRNA molecules that have recently become a research hotspot in the field of RNA. They are endogenous RNAs formed by alternative splicing and are widely distributed in eukaryotic cells. The structure of circRNA molecules is relatively stable and is different from that of linear RNA molecules because it is a closed circular molecule without 5′ and 3′ ends and is not affected by exonucleases^72^. Previous studies have proposed that circRNAs can serve as effective miRNA sponges, as they contain conserved miRNA targets, competitively inhibiting miRNA regulation of downstream target gene expression^73–75^. Moreover, studies have confirmed that circRNAs can be enriched in exosomes^11,76^. These molecules have various functions in the body and participate in many physiological processes, such as the proliferation and invasion of tumor cells and the progression of other diseases^76^.

### Exosomal circRNAs in tumor growth

It has been reported that exosomal circRNAs function mainly by sponging miRNAs. A study from Yongchao Li et al. demonstrated that the circRNA FMN2 was highly abundant in the serum of CRC patients. Functional experiments and bioinformatics analysis indicated that the circRNA FMN2 promotes CRC proliferation via the miR-1182/hTERT signaling pathway^77^. In another study, the circRNA IFT80 was found to be significantly increased in the serum and cells of CRC patients. Silencing IFT80 inhibited CRC cell proliferation in vitro and in vivo. Mechanistically, the exosomal circRNA IFT80 exerts its function through the miR-1236-3p/HOXB7 axis^78^. In addition, Zhang et al. found that the adipose-secreted circRNA circ-DB regulates deubiquitination in HCC by controlling miR-34a and deubiquitination-related USP7, thereby promoting cell growth^79^. Similarly, a study using a xenograft mouse model of liver cancer cells revealed that tumor-derived exosomal circRNAs in serum were related to tumor mass. Exosomal circRNAs can maintain biological activity and abolish miRNA-induced growth inhibition in recipient cells^80^. In gastric cancer (GC), one study proved that knockdown of the circRNA NRIP1 can suppress the growth of GC cells. In terms of the mechanism, NRIP1 may act as a suppressor by sponging miR-149-5p, but this speculation needs further demonstration^81^. In another article, Xie et al. demonstrated that exosomal circSHKBP1 regulates the miR-582-3p/HUR/VEGF pathway, suppresses HSP90 degradation, and promotes GC progression^82^.

### Exosomal circRNAs in tumor metastasis

Tumor metastasis requires not only growth and invasion abilities but also the ability of cancer cells to cross the endothelial barrier. The process of EMT is also regulated by many circRNAs, which indicates that circRNAs may have an important role in EMT-related processes, including metastasis^83^. At present, our understanding of the function of exosomal circRNAs in tumor metastasis and their molecular mechanisms is limited. A study pointed out that circ-RanGAP1 was significantly elevated in the tissues and plasma exosomes of patients with GC, which was related to the late stage of tumor node metastasis. A mechanistic study showed that circ-RanGAP1 elevated the expression levels of VEGF-A (VEGFA) by sponging miR-877-3p^84^. Similarly, the expression of exosomal circ-FECR was increased in small cell lung cancer (SCLC), which was associated with its metastasis^85^. Moreover, an analysis of the association between the levels of the circRNA ciRS-7 and the clinicopathological characteristics of HCC patients indicated that the levels of ciRS-7 were highly associated with hepatic microvascular invasion^86^. Intriguingly, circRNAs could be induced by hypoxia, which is one of the important properties of solid tumors. One article suggested that hypoxia-derived exosomal circ-133 was transported into normoxic cancer cells and promoted cell migration via the miR-133a/GEF-H1/RhoA axis^87^.

### Exosomal circRNAs in tumor angiogenesis

CircRNAs have recently been regarded as a vital factor in cancer progression; however, the function of exosomal circRNAs in tumor angiogenesis has not been explored clearly. Specifically, few studies have examined the role of exosomal circRNAs in tumor angiogenesis, resulting in a largely unknown understanding of their underlying mechanisms, which urgently requires further exploration. Currently, only one study has shown that coculture of HUVECs and exosomes derived from HCC cell lines significantly enhances HUVEC angiogenesis and tumor metastasis. Further study found that exosomal circRNA-100,338, which was highly expressed in both highly metastatic HCC cells and their secreted exosomes, mediates angiogenesis^88^.

### Exosomal circRNAs in tumor chemoresistance

As various potential mechanisms are involved in the progression of drug resistance, exosomes have attracted extensive attention as a new therapeutic approach. Recently, studies have indicated that circRNAs can regulate chemotherapy resistance in many kinds of cancers^89^. In SCLC, exosomal circRNA-FECR1 is considered to be important for chemoresistance. Data revealed that exosomal circRNA-FECR1 was highly abundant in the serum of SCLC patients compared with that of healthy controls and was clinically associated with poor survival and chemotherapy response^85^. Moreover, in glioma, one study revealed that the expression of exosomal circRNA NFIX was significantly increased in the serum of patients with temozolomide resistance. The exosomal circRNA NFIX mediates the resistance of gliomas to temozolomide at least in part by sponging miR-132^90^. Regarding CRC, a study suggested that PKM2 expression levels in CRC cells are heterogeneous; that is, PKM2 is abundant in oxaliplatin-resistant cells and relatively low in drug-sensitive cells. A mechanistic study indicated that once transferred from resistant cells to sensitive cells, the circRNA hsa-circ-0005963 could transmit oxaliplatin resistance by sponging miR-122 and upregulating PKM2^91^. Furthermore, a microarray analysis of exosomal RNAs from chemoresistant and chemosensitive CRC cells was performed by Hon et al. to identify key exosomal RNAs associated with chemoresistant CRC. The results showed that exosomal circ_0000338 may have dual regulatory roles in chemoresistant CRC^92^. Recently, another study revealed that HCC-derived exosomal circUHRF1 contributes to immunosuppression by inducing NK cell dysfunction, thus driving resistance to anti-PD1 immunosuppression^93^.

Overall, abnormal expression of exosomal circRNAs has been found in various types of cancer. By gain- and loss-of-function assays, researchers have identified the role of exosomal circRNAs in the progression of cancer, which provides a better understanding of the relationship between exosomal circRNAs and cancer^94–103^ (Table 1). However, the mechanism of exosomal circRNAs in cancer deserves further study.

**Table 1:** Generalization of exosomal circRNAs in tumor progression.

Exosomal circRNA	Cancer type	Function	Reference
Circ-PDE8A	Pancreatic ductal adenocarcinoma	Promoting cell invasion	^94^
Circ-IARS	Pancreatic ductal adenocarcinoma	Facilitating cell invasion and metastasis	^95^
CircRNA-100284	Hepatocellular carcinoma	Accelerating cell cycle and promoting cell proliferation	^96^
Hsa_circ_0051443	Hepatocellular carcinoma	Inducing cell cycle arrest and promoting cell apoptosis	^97^
Circ-PTGR1	Hepatocellular carcinoma	Promoting cell metastasis	^98^
Circ-0000284	Cholangiocarcinoma	Enhancing cell migration, invasion and proliferation	^99^
Circ-WHSC1	Ovarian cancer	Increasing cell proliferation, migration and invasion, and inhibiting cell apoptosis	^100^
Circ-NT5E	Glioblastoma	Inhibiting cell proliferation, migration, and invasion	^101^
Circ-RASSF2	Laryngeal squamous cell carcinoma	Accelerating cell proliferation and migration	^102^
Circ-RTN4	Colorectal cancer	regulating chemotherapy resistance	^103^

## The roles of lncRNAs in cancer

### Background on lncRNAs

LncRNAs generally refer to RNA transcripts longer than 200 nucleotides (Table 2). It was originally believed that lncRNAs were transcriptional byproducts of RNA polymerase II that could not be translated into proteins and had no biological functions. However, increasing amounts of evidence have proven that they actually participate in many biological reactions, such as chromatin remodeling, histone modification and X chromosome inactivation. In fact, by directly or indirectly interfering with gene expression in a variety of cancers, lncRNAs were demonstrated to play important roles in multiple biological processes, including cell growth, differentiation, and proliferation^104^. Notably, lncRNAs can bind to various biological macromolecules in cells, such as DNA, RNA, and proteins^105^. Similarly, via exosomes, lncRNAs can be transported into body fluids. Interestingly, some lncRNAs are enriched in exosomes, while others are virtually absent, suggesting that some lncRNAs are selectively classified into exosomes. Exosomal lncRNAs account for approximately 3% of the total RNA of exosomal species.

**Table 2:** Exosomal lncRNAs associated with tumor progression.

Function	Exosomal lncRNA		Cancer	Mechanism	Reference
Growth		UFC1	Non-small-cell lung cancer	Inhibiting PTEN expression via EZH2-mediated epigenetic silencing	^109^
		RUNX2-AS1	Multiple myeloma	Repressing the osteogenesis of MSCs.	^110^
		UCA1	Bladder cancer	Downregulating E-cadherin; upregulating vimentin and MMP9	^111^
Metastasis		ROR	Thyroid cancer		^116^
		MMP2-2	Lung cancer	Upregulating MMP2	^117^
		91H	Colorectal cancer	Upregulating HNRNPK expression	^118^
		RPPH1	Colorectal cancer	mediating macrophage M2 polarization	^119^
Chemoresistance		VLDLR	Hepatocellular carcinoma	Upregulating ABC-G2	^134^
		ROR	Hepatocellular carcinoma	Inhibiting the p53 signaling pathway	^135^
		HNF1A-AS1	cervical cancer	upregulating the expression of TUFT1 and downregulating miR-34b.	^136^

### Exosomal lncRNAs in tumor growth

Many exosomal lncRNAs are involved in tumorigenesis and tumor growth. Some of them are downregulated in pathological samples to promote tumor growth. A study by Zhang et al. detected exosomal lncRNA-H19 content in the serum of healthy subjects and patients with pituitary tumors and found that the expression of lncRNA-H19 was significantly reduced in tumor patients. Further studies demonstrated that exosomal lncRNA-H19 could suppress distal tumor cell growth by inhibiting 4E-BP1 phosphorylation^106^. Additionally, the lncRNA SENP3-EIF4A1 was found to be significantly decreased in the serum of HCC patients. Research has shown that SENP3-EIF4A1 derived from exosomes could inhibit tumor growth in vivo and modulate the levels of ZFP36 ring finger protein (ZFP36) by competitively binding to miR-9-5p^84^. There are, of course, some lncRNAs that are abnormally upregulated in pathological samples. For example, the expression of the lncRNA prostate cancer-expressed EZH2-associated transcript (PCSEAT) was suggested to be specifically upregulated in prostate cancer (PCa)^107^. Similarly, the lncRNA BCRT1, a lncRNA that can remarkably suppress cancer progression both in vivo and in vitro, was markedly increased in BC tissues. In terms of the mechanism, lncRNA BCRT1 was able to competitively bind with miR-1303 and inhibit the degradation of the target gene polypyrimidine tract-binding protein 3 (PTBP3), which can promote the progression of BC^108^. These clues all suggest that exosomal lncRNAs could regulate cell growth by targeting specific factors or cell pathways associated with cell proliferation. However, the research on exosomal lncRNAs and tumor growth is far more abundant^109–111^.

### Exosomal lncRNAs in tumor metastasis

Recently, research on exosomal lncRNAs has made rapid progress. For example, Jin et al. suggested that the lncRNA TIRY was able to promote tumor metastasis by enhancing the EMT process in oral cancer^112^. Similarly, Q Zhang et al. demonstrated that SKOV3-secreted exosomes suppressed the phosphatase and tensin homolog (PTEN)/AKT signaling pathway through the transfer of the lncRNA FAL1, thereby inhibiting OC cell metastasis in vitro and in vivo^113^. In addition, in a study of ESCC progression, Li et al. found that ESCC tissues showed high expression of lncRNA-ZFAS1 and low expression of miR-124. Gene silencing experiments confirmed that the silencing of ZNFX1 antisense RNA 1 (ZFAS1) inhibited the development of ESCC^79^. Additionally, exosomal lncRNA-MALAT1 was found to be upregulated in NSCLC^114^. In vitro studies have shown that exosomal lncRNA-MALAT1 facilitates tumor proliferation and metastasis and inhibits tumor apoptosis. Mechanistically, it mainly exerts its function by modulating the levels of cyclin D1 and cyclin D2 in NSCLC. In contrast, exosomal lncRNA-PTENP1, an exosome with reduced expression in bladder cancer patients, was shown to inhibit the biological malignancy of bladder cancer cells by increasing apoptosis rates and reducing cell invasion and migration capacity^115^. Not surprisingly, other similar studies have revealed the role of exosomal lncRNAs in tumor metastasis^116–119^.

### Exosomal lncRNAs in tumor angiogenesis

An increasing number of studies have shown that unusual lincRNA expression has an important effect on tumor progression, including angiogenesis and tumor development^120^. Conigliaro et al. showed that exosomes that are high in lincRNA H19 secreted by CD90^+^ LCCs could be absorbed by endothelial cells and promote angiogenic phenotypes and intercellular adhesion^121^. In terms of the mechanism, functional studies have demonstrated that exosomal lincRNA H19 functions by managing the levels of VEGF and VEGF receptor 1 (VEGFR1). Dynamic changes in endothelial cells are an important feature of angiogenesis^122^. However, many genes are involved in endothelial cell function^123^. The study of Lang et al. suggested that exosomal lincRNA-POU3F3 was abnormally elevated in glioma and was associated with advanced tumors. Further mechanistic studies revealed that glioma cells were able to transport lincRNA-POU3F3 to endothelial cells in the form of exosomes and induce angiogenesis by modulating angiogenic factor levels^124^. Additionally, in another study of glioma, exosomes from U87-MG cells that were enriched in lincRNA-CCAT2 were also shown to be internalized by HUVECs, thereby promoting the angiogenesis of HUVECs^125^. Furthermore, in lung cancer cells, the exosomal lncRNA GAS5 could bind to miRNA-29-3P in competition with PTEN, thereby inhibiting phosphatidylinositol 3-kinase (PI3K) levels and promoting HUVEC proliferation and tube formation^126^. In general, tumor cells are able to transfer lncRNAs to endothelial cells through exosomes, and these lncRNAs can promote the production of angiogenesis-related factors and molecules, thus promoting tumor angiogenesis.

### Exosomal lncRNAs in tumor chemoresistance

Recently, the relationship between exosomal lncRNAs and tumor chemoresistance has received increasing attention. A study by Wang et al. showed that exosomal lncRNA H19 can be delivered to doxorubicin-sensitive BC cells, leading to the dissemination of doxorubicin resistance^127^. In another study on BC, a significant increase in exosomal lncRNA-SNHG1 was found in trastuzumab-resistant cells^128^. Functional experimentation showed that lncRNA-SNHG14 knockdown could effectively promote the efficacy of trastuzumab. According to the Signal Transduction Reporter Array, the lncRNA SNHG14 was likely to generate resistance by targeting the apoptosis regulator Bcl-2/Bax signaling pathways. Likewise, the expression levels of lncRNA-UCA1 were notably upregulated in BCC patients with tamoxifen resistance^129^.

In addition to BCCs, exosomal lncRNAs have also been extensively studied in other tumors. For example, the exosomal lncRNA HOTTIP was shown to contribute to the cisplatin resistance of GC cells by regulating the miR-218/high mobility group AT-hook 1 (HMGA1) axis^130^. Additionally, in a study of erlotinib resistance in NSCLC, Wei Zhang et al. revealed that in erlotinib-resistant NSCLC cells, the expression of the exosomal lncRNA RP11-838N2.4 was elevated^131^. Likewise, in a study on ESCC resistance, Min Kang et al. demonstrated that the levels of the exosomal lncRNA PART1 were upregulated in ESCC cells, which generated gefitinib resistance. Experimental results have shown that the lncRNA PART1 promotes the generation of resistance by regulating the miR-129/Bcl-2 pathway^132^. For renal cell carcinoma (RCC), lncRNA profile analysis and sequence screening indicated that lncRNA-ARSR was highly enriched in sunitinib-resistant RCC cells. Further experiments showed that lncRNA-ARSR promoted RCC cell resistance to sunitinib by competitively binding miR-34/miR-449 to promote AXL receptor tyrosine kinase (AXL) and c-MET expression^133^. However, more studies on the relationship between exosomal lncRNAs and chemoresistance are ongoing, which will help us better understand the mechanism of tumor resistance^134–136^.

## Other exosomal ncRNAs in cancer

### snRNAs

snRNAs are the major component of RNA spliceosomes in eukaryotic posttranscriptional processing and participate in the processing of mRNA precursors. Endogenous variation in snRNAs could potentially enable these RNAs to regulate alternative splicing to drive genetic, dysplastic, and neoplastic disease^137,138^ However, there are a very limited number of studies exploring the roles of exosomal snRNAs in cancer progression. One study revealed that exosomal snRNAs in cancer cells mediate the activation of Toll-like receptor 3, therefore inducing chemokine secretion, which contributes to neutrophil mobilization and recruitment, leading to the generation of the lung premetastatic niche and further metastasis^139^. In addition, another recent study suggested that exosomal snRNA U1, U2, and U5 levels were significantly decreased in patients with lung cancer compared with early-stage patients, indicating their potential as biomarkers in lung cancer^140^. Nevertheless, the function of secreted snRNAs in cancer has largely not been explored, and more attention should be given to this topic.

### snoRNAs

snoRNAs are a class of small molecular ncRNAs widely distributed in the nucleolus of eukaryotic cells. They have conserved structural elements and can be divided into three categories: box C/D snoRNA, box H/ACA snoRNA, and MRPRNA. SnoRNAs have a significant influence on the posttranscriptional modification of rRNA. However, few studies on exosomal snoRNAs in cancer have been reported to date^141^. Only one study revealed that the snoRNAs SNORD33, SNORD66 and SNORD76 showed higher expression levels in NSCLC patients than in healthy individuals^142^. Such a situation raises many questions, such as what roles do snoRNAs play in cancer and how do they function? We believe that an in-depth understanding of exosomal snoRNA in tumors may contribute to the design of cancer-diagnostic and cancer-prognostic tools. Obviously, more attention should be given to this subject.

## Clinical implications of exosomal ncRNAs in cancer treatment

The poor prognosis of some cancer treatments is largely due to the detection of late-stage disease, partly caused by the deficiency of suitable biomarkers. As discussed above, exosomal ncRNAs have a key effect on the pathological process of various cancers, thus exosomal ncRNAs associated with the generation and progression of tumors can potentially be used as diagnostic, prognostic, and predictive biomarkers for cancer. In addition, exosomes are present in a variety of body fluids, such as blood, urine, saliva, breast milk, and ascites^143–147^, and this makes it easy to collect them and detect their levels. Therefore, they can be used as a liquid tool for noninvasive clinical testing.

To date, the most widely studied exosomal ncRNAs are exosomal miRNAs. Multiple studies have shown that exosomal miRNAs have the potential to be used as tools for the diagnosis of various cancers. For example, a study revealed the underlying role of exosomal miR-373 as a biomarker for aggressive tumors. Its identification can be used for the diagnosis of BC^53^. In another study, miRNA expression profiles were conducted to identify exosomal miRNAs that could distinguish malignant cancer from benign disease. The results showed that eight tumor-specific exosomal miRNAs (miR-21, miR-141, miR-200a, miR-200c, miR-200b, miR-203, miR-205, and miR-214) were significantly distinct in profiles between benign disease and OC, and they could be used as surrogate diagnostic markers for biopsy profiling^148^. Moreover, Cazzoli et al. suggested that several exosomal miRNAs (miR‐200b-5p, miR-378a, miR-139-5p, and miR-379) could distinguish nodules and nonnodules and that other exosomal miRNAs (miR-629, miR-30a-3p, miR-100, miR-200b-5p, miR-154-3p, and miR-151a-5p) could discriminate granulomas from lung adenocarcinoma, indicating their diagnostic value in lung cancer^149^. Of course, in addition to being diagnostic biomarkers, exosomal miRNAs have also been shown to be used as prognostic biomarkers for cancer. In a study by Xiaoyi Huang et al., it was shown that high levels of two plasma exosomal miRNAs (miR-1290 and miR-375) were significantly associated with poor overall survival in castration-resistant PCa, indicating their potential as prognostic biomarkers^150^. Similarly, compared to primary tumors, exosomal miR-21 and miR-155 were significantly increased in recurrent tumors, which suggested their potential as prognostic biomarkers^151^. Interestingly, exosomal miRNA-21 has also been shown to be related to the TNM stage of HCC, and its high level was associated with poor prognosis in CRC^152,153^. These distinct exosomal miRNAs function by regulating differential signaling pathways and may serve as promising targets for cancer therapy.

Apart from exosomal miRNAs, some researchers have suggested that exosomal lncRNAs could act as a diagnostic tool in cancer. A study showed that the levels of the exosomal lncRNAs ENSG00000258332.1 and LINC00635 were significantly increased in HCC patients compared with chronic hepatitis B patients^154^. Studies have shed light on lncRNAs from exosomes as diagnostic biomarkers for HCC cells. Similarly, an analysis demonstrated that higher expression levels of exosomal lncRNA ATB can serve as a prediction tool in cancer prognosis, since the overall survival of patients in the exosomal lncRNA ATB high expression groups was shorter^152^. Another study indicated that elevated levels of the lncRNA MIAT were related to shorter survival in GC patients^155^. Recently, based on public databases and the integration of bioinformatics analyses, exosomal lncRNA BCRT1 was demonstrated to be a potential biomarker and therapeutic target for BC^108^.

Compared with other ncRNAs that exhibit the potential to function as cancer biomarkers, circRNAs with a more stable circular structure seem to have more potential as tumor biomarkers. A study showed that the increased expression of exosomal circPRMT5 was associated with advanced clinical stage and poor survival in patients with urothelial carcinoma of bladder cancer patients, and data demonstrated that the exosomal circRNA PRMT5 exerts its protumor effect by sponging miR-30c. An analysis suggested that the exosomal circRNA PRMT5 may act as an important therapeutic target for patients^156^. In another study, exosomal circRNA FNDC3B levels were elevated in papillary thyroid carcinoma (PTC) tissues and cell lines. Guojun Wu et al. showed that the exosomal circRNA FNDC3B regulated PTC progression via the miR-1178/TLR4 axis and suggested that it was likely to be a potential target for the treatment of PTC^157^. Furthermore, hsa_circ_0000419, hsa_circ_0065149, and circ-KIAA1244 were suggested to serve as potential diagnostic and/or prognostic biomarkers for GC^158–160^. Exosomal circRNAs represent promising biomarkers and therapeutic targets in cancer. Nonetheless, the study of the role of exosomal circRNAs in cancer is still ongoing.

However, for exosomal snRNAs, only one study revealed that the decreased levels of exosomal snRNA U1, U2 and U5 were correlated with lung cancer progression, possessing favorable diagnostic efficiency, suggesting that the aberrant expression of snRNA U1, U2, and U5 could act as novel biomarkers for lung cancer^140^.

With developments in biotechnology, exosomes can replace synthetic nanoparticles. As natural nanoparticles, exosomes have better biocompatibility and lower biological toxicity and immunogenicity; therefore, exosomal ncRNAs are a potential choice for novel cancer treatments. A study has shown that synthesized sphingosine kinase 2 (Sphk2) siRNA-loaded nanoparticles could reduce exosomal miR-21 levels and inhibit tumor migration in HCC cells^161^. In addition, Li et al. used BMSCs to obtain modified exosomes to deliver GRP78 siRNA to HCC. The results showed that GRP78 siRNA combined with sorafenib can target glucose-regulated protein 78 (GRP78), which is overexpressed in sorafenib-resistant HCC and sensitizes sorafenib-resistant HCC to sorafenib, thus reversing drug resistance^162^. In addition, Chunhui Wang et al. found that transient receptor potential polycystic 2 (TRPP2) expression was increased in laryngeal squamous cell carcinoma (LSCC) and promoted the metastatic ability of cancer cells by regulating the EMT process. By targeting TRPP2 with the exosome/TRPP2 siRNA complex, the expression of N-cadherin and Vimentin in LSCC cells was notably reduced, while the expression of E-cadherin was elevated. This revealed that the exosome/TRPP2 siRNA complex could effectively inhibit the metastasis of LSCC^163^. These studies reveal a novel cancer therapeutic method involving the ablation of oncogenic miRNAs from exosomes.

**Fig. 3:**
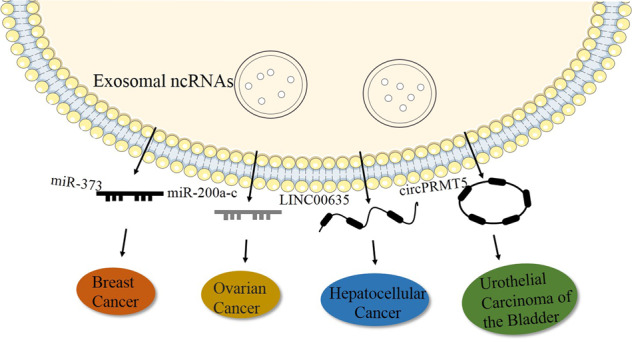
Different exosomal ncRNAs can act as diagnostic factors in various cancers. Exosomal miR-373 and miR-200a-c could serve as diagnostic factors for breast cancer and ovarian cancer separately. Exosomal linc00635 could serve as diagnostic factor for hepatocellular cancer. Exosomal cicrPRMT5 could serve as diagnostic factor for urothelial carcinoma of the bladder.

Generally, exosomal ncRNAs have great prospects in the applications of cancer therapy due to their structural stability, which effectively prevents the digestion and degradation of RNA by related enzymes. Given the biocompatibility of exosomes, they can be engineered to deliver therapeutic factors (such as RNA, proteins and drugs) to target cells for therapeutic applications. In addition, based on the targeting of exosome transport, they can be used as carriers of drugs in vivo, which has great potential in clinical applications such as targeted therapy for tumors and disease course monitoring (Fig. 3).

## Discussion

Exosomes exert their vital functions in many pathophysiological processes, such as inflammatory reactions, immunoregulation, tumor progression, tumor invasion, and metastasis. NcRNAs exist in different intercellular compartments and participate in different cellular reactions. They can also be identified in biological fluids and are called circulating ncRNAs, and these ncRNAs can be detected in exosomes.

Specifically, in this review, we highlighted the biological characteristics of exosomes and presented a comprehensive update on the functions of exosomal miRNAs, circRNAs, and lncRNAs in tumor growth, metastasis, angiogenesis and tumor chemoresistance. The role of these ncRNAs may be tumor supportive or tumor suppressive in different cancers. Therefore, considerable focus has shifted to the cancer-related signatures of these ncRNAs.

We also described the therapeutic implications of exosomal ncRNAs in cancer treatment, suggesting that many ncRNAs may be regarded as predictive markers. Due to abnormal chromatin remodeling and alternative splicing programs in cancer, lncRNAs and circRNAs can be extremely cancer type-specific. Thus, exosomal lncRNAs and circRNAs can be useful cancer-specific biomarkers. Because exosomal ncRNAs have great diagnostic and therapeutic potential, a large number of studies indicate that further exploration is needed, but there are still many challenges. More research on the roles and regulation of the identified ncRNAs is needed to understand their molecular mechanism.
